# Possible Role of Crystal-Bearing Cells in Tomato Fertility and Formation of Seedless Fruits

**DOI:** 10.3390/ijms21249480

**Published:** 2020-12-13

**Authors:** Ekaterina N. Baranova, Inna A. Chaban, Ludmila V. Kurenina, Ludmila N. Konovalova, Natalia V. Varlamova, Marat R. Khaliluev, Alexander A. Gulevich

**Affiliations:** 1 Plant Protection Laboratory, N.V. Tsitsin Main Botanical Garden of Russian Academy of Sciences, 127276 Moscow, Russia; konovalova-lu@yandex.ru; 2 Cell Biology Laboratory, All-Russian Scientific Research Institute of Agricultural Biotechnology, 127550 Moscow, Russia; inna_chaban@rambler.ru; 3 Plant Cell Engineering Laboratory, All-Russian Scientific Research Institute of Agricultural Biotechnology, 127550 Moscow, Russia; ludmila.kur2208@gmail.com (L.V.K.); nv_varlamova@rambler.ru (N.V.V.); marat131084@rambler.ru (M.R.K.); 4 Agronomy and Biotechnology Faculty, Moscow Timiryazev Agricultural Academy, Russian State Agrarian University, Timiryazevskaya 49, 127550 Moscow, Russia

**Keywords:** calcium oxalate, mesophyll ultrastructure, anther structure, anther dehiscence, crystal-bearing cells, pollen fertility, programed death, transgenic plant

## Abstract

Crystal-bearing cells or idioblasts, which deposit calcium oxalate, are located in various tissues and organs of many plant species. The functional significance of their formation is currently unclear. Idioblasts in the leaf parenchyma and the development of crystal-bearing cells in the anther tissues of transgenic tomato plants (*Solanum lycopersicon* L.), expressing the heterologous *FeSOD* gene and which showed a decrease in fertility, were studied by transmission and scanning electron microscopy. The amount of calcium oxalate crystals was found to increase significantly in the transgenic plants compared to the wild type (WT) ones in idioblasts and crystal-bearing cells of the upper part of the anther. At the same time, changes in the size and shape of the crystals and their location in anther organs were noted. It seems that the interruption in the break of the anther stomium in transgenic plants was associated with the formation and cell death regulation of a specialized group of crystal-bearing cells. This disturbance caused an increase in the pool of these cells and their localization in the upper part of the anther, where rupture is initiated. Perturbations were also noted in the lower part of the anther in transgenic plants, where the amount of calcium oxalate crystals in crystal-bearing cells was reduced that was accompanied by disturbances in the morphology of pollen grains. Thus, the induction of the formation of crystal-bearing cells and calcium oxalate crystals can have multidirectional effects, contributing to the regulation of oxalate metabolism in the generative and vegetative organs and preventing fertility when the ROS balance changes, in particular, during oxidative stresses accompanying most abiotic and biotic environmental factors.

## 1. Introduction

In plants, the formation of calcium oxalate crystals is a regulated process that accompanies specific transformations in tissue cells, which are characterized by the presence of these crystals [1,2]. In various families of higher plants, calcium oxalate occurs quite frequently and is observed in most plant tissues and organs in the form of intracellular and extracellular deposits [3]. Plants produce and deposit oxalate up of 3–80% of the total dry weight [4]. In this case, up to 90% of the total content of plant calcium can be in the form of oxalate crystals [5]. Intracellular crystals are fairly common in specialized mesophyll cells called idioblasts [6]. These cells have an altered organelle ultrastructure [7], and despite being located in actively photosynthetic tissue, have underdeveloped systems of thylakoids and granae.

The formation of oxalate crystals is regulated by the biosynthesis of oxalic acid and calcium metabolism and has specific features in different types of cells and tissues [2]. Several varieties of calcium oxalate crystals have been described and characterized as raphids, amorphous crystals and druses [8]. There is now strong evidence that the scavenging of oxalic acid and excess Ca^2+^ in the form of crystals can have the following functions: regulation of Ca^2+^ balance [1], protection of plants from pathogens [9] and detoxification [10,11,12], reducing animal consumption [13,14], tissue support for plant rigidity, and light collection and reflection [5].

Calcium oxalate crystals play an important role in the generative development of many higher plants [15]. They provide a sophisticated anther dehiscence mechanism to ensure the timely release of pollen [16,17]. The appearance of calcium oxalate crystals precedes the opening of the anther and ensures the formation of a hole in its upper third and dehiscence of the anther simultaneously with lysis of the crystal-bearing parenchyma [16,18]. Disruption of oxalate formation in this specialized zone leads to blockage of the conjunction of paired pollen sacs [18], impaired fertility accompanied by defects in the development of anther ridges, dehiscence [19] and pollen yield [20]. It is shown that the growth of prismatic crystals and druses may occur by the formation of crystal-framing lamellae or chambers with the deposition of Ca, Mg, and/or Si [21].

There are many examples of morphological abnormalities in anthers accompanied by impaired fertility. This is especially true for self-pollinated plants such as tomato [22]. Anther dehiscence failure and morphological defects can be caused by unfavorable environmental conditions [23], pathogens or genetic disorders [24,25].

The enlargement of fruits, as a rule, is the most valuable and in many respects defining trait. As a result, there is a significant modification of the flower and ovary, accompanied by a change in the number of petals, anthers, as well as the shape of the ovary and the number of locular cavities in the ovary and fruit [26,27]. Tomato flowers are collected in simple or complex (branched) inflorescences, bisexual. Usually they have a five-membered yellow corolla with a diameter of about 1 cm and five narrow stamens accreted into a conic tube. There is a pistil inside it. Anthers have two thecae, with two pollen sacs in both anther thecae. They open with internal slits through which pollen spills out and gets on the stigma of the pistil. In modern varieties, the number of ovary and fruit locular cavities, the number of stamens, petals and sepals can vary significantly, and increases with an enlargement in fruit size and number of cavities. Small-fruited varieties with large inflorescences are an exception.

Fertility of fruits in plant varieties with large fruits, as well as of various hybrids and transgenic forms, can be significantly reduced [28], and in some cases leads to the formation of parthenocarpic fruits [29], and this is a problem to obtain seeds from such plants.

Reactive oxygen species caused by metabolic disorders associated with external and internal characteristics of the reaction of cells and tissues cause various damages at the cellular level, such as micro- and macroautophagy, as well as programed cell death. Modification of the enzyme pool and activity associated with reactive oxygen species (ROS) neutralization has been described for many types of plant injuries, ranging from mechanical stress, possibly accompanying interrelationships between growth processes, and ending with infectious damages caused by fungi, bacteria, insects, and nematodes. Thus, nonspecific H_2_O_2_ increase, caused by heterologous expression of Fe-dependent superoxide dismutase in one of the transgenic tomato lines [30], was not accompanied by the inactivating effect of peroxide scavenging enzymes. Such a detoxification of hydrogen peroxide seemed to us to be a successful model for revealing the role of ROS in impaired development of the male gametophyte and, in particular, the formation of oxalate crystals in the specialized zone of the barrier between pollen sacs inside of anther. The functional role of oxalates in maintaining the fertility of the male gametophyte is of interest to many researchers [31,32]. Various varieties and forms with pronounced male sterility served as model plants, but the use of transgenic plants was proposed by us for the first time.

The aim of this work was to identify the differences in the formation of idioblasts in the leaf mesophyll and parenchymatous crystal-bearing cells in the barrier zone between pollen sacs in the anther of transgenic tomato plants, which expressed Fe-dependent superoxide dismutase, causing impaired fertility and an accompanying increase in resistance to biogenic and abiotic damage [33,34].

## 2. Results

### 2.1. Characteristics of Flowers, Anthers and Pollen Structure of Transgenic and WT Plants

**Figure 1:**
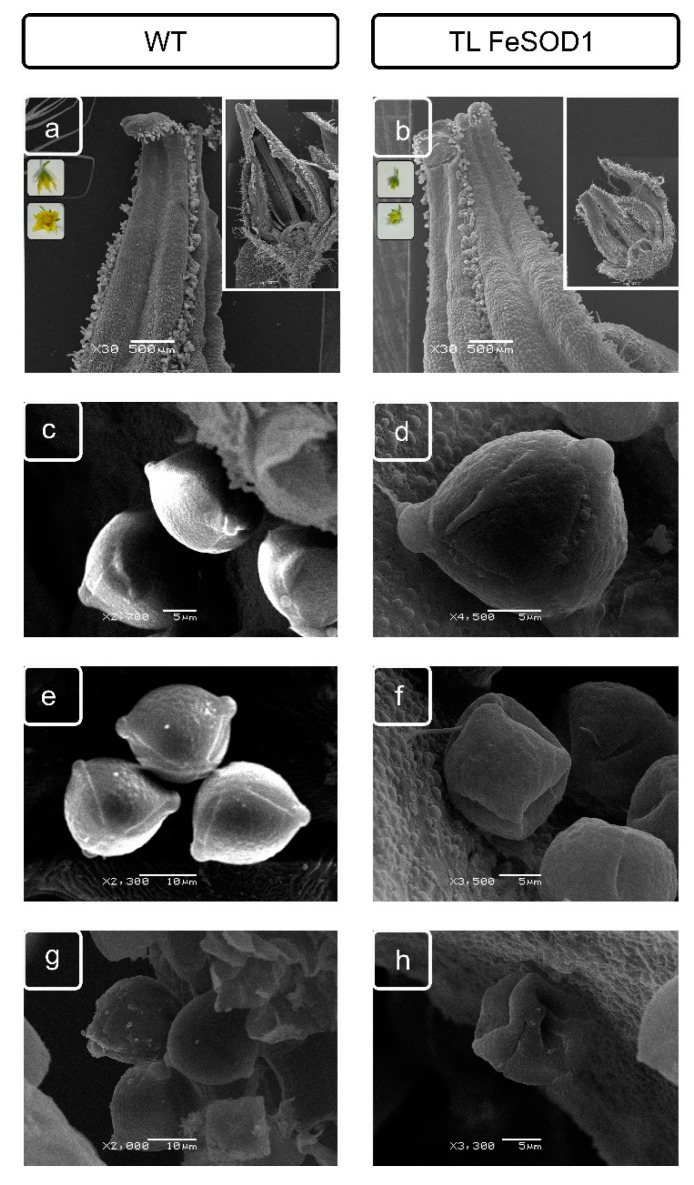
Transmission scanning microscopy of flowers (**a**,**b,** inserts), formed anthers (**a**,**b**), and pollen (general appearance—(**c**,**d**); exine and germpore of pollen grain—(**e**–**h**) from WT (**a**,**c**,**e**,**g**) and transgenic (**b**,**d**,**f**,**h**) tomato lines.

The tomato transgenic line 6, overexpressing the gene of Fe-dependent superoxide dismutase (FeSOD) [30], had obvious problems with fertility; most of the flowers were smaller than the WT (Figure 1a) and fell off after flowering. The ripening fruits were smaller than in the WT tomatoes, and had single seeds or were parthenocarpic. The flowers developed by this line were also smaller and less intense in color. Their anthers had a shape different from the WT type (Figure 1a) and were half the height of the WT flower stamens (Figure 1b, color insert). The anther wall in the tomato flower at the end of the microsporogenesis stage consists of the tapetum layer, which ensures the development of pollen and the formation of its envelopes; adjacent cells of the middle layer, represented by one or two layers of cells; a single-layer fibrous endothecium located above it; and the epidermal layer on the anther surface (Figure S1). This accompanied significant differences in the morphology of anthers and pollen, indicating a cumulative disruption in the development of vegetative (providing development) and generative tissues of the male gametophyte with a change in the associated metabolism caused by overexpression of FeSOD (Figure 1b,d,f,h), in particular, deformation of some pollen grains.

**Figure 2:**
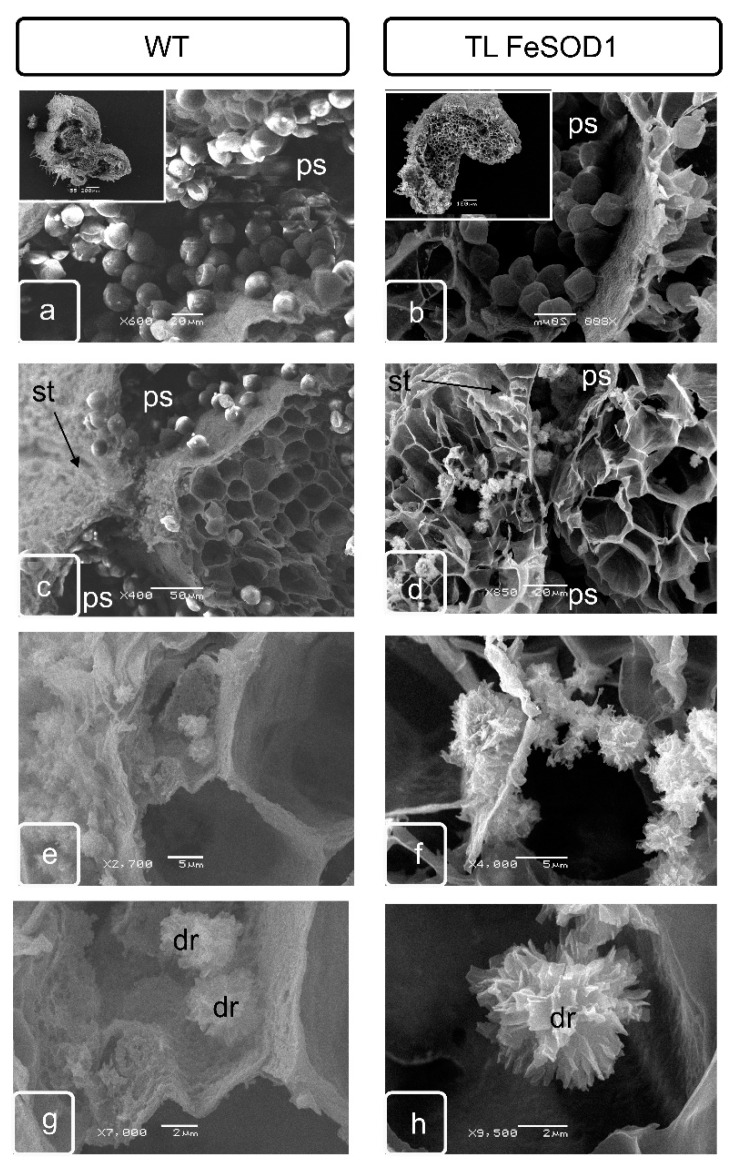
Scanning electron microscopy of pollen sac cells and stomium zone from WT (**a**,**c**,**e**,**g**) and transgenic (**b**,**d**,**f**,**h**) tomato plants. Ps—pollen sac; st—stomium; dr—druse.

**Figure 3:**
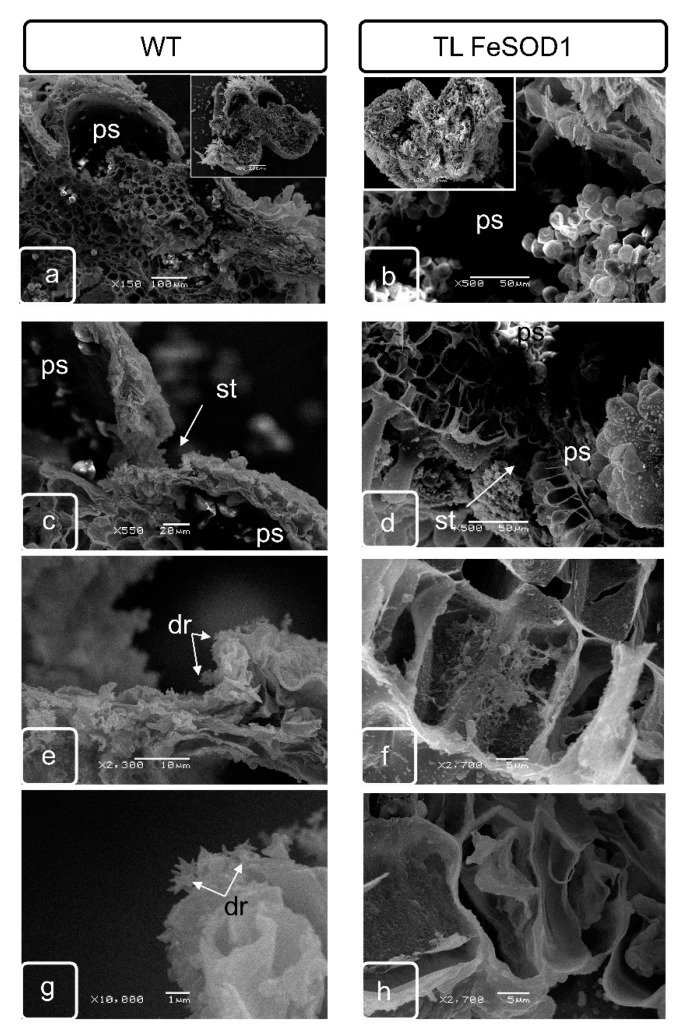
Scanning electron microscopy of cross sections at the top of anther. Pollen sac with pollen grains (**a**,**b**). General view of the cross section (inserts on (**a**,**b**)) WT (**a**,**c**,**e**,**g**) and transgenic (**b**,**d**,**f**,**h**) tomato plants. The junction site of the pollen sacs in the stomium zone characterizes by the formation of a crystal-bearing parenchyma, which contain the calcium oxalate crystals. The site of mass formation of calcium oxalate druses (**e**,**g**). Ps—pollen sac; st—stomium; dr—druse.

**Figure 4:**
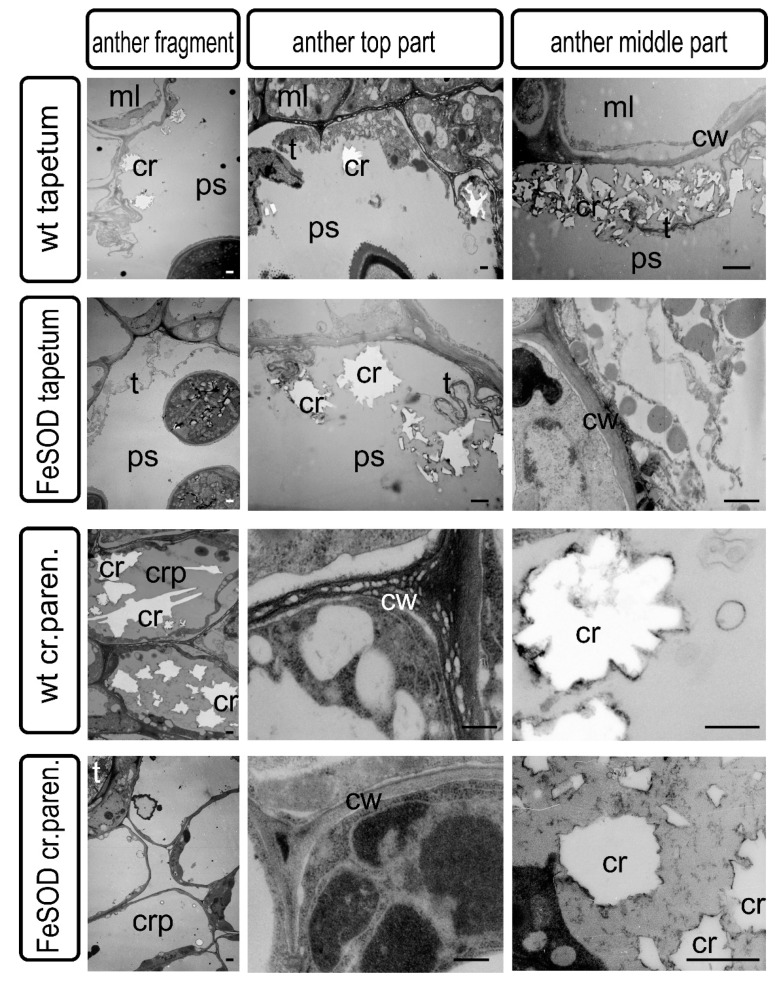
Transmission electron microscopy of cross sections of the anther tissue. The **top** two rows represent tapetum cells in the anthers of WT and transgenic tomato plants. The **bottom** two rows represent crystal-bearing cells in the anthers of WT and transgenic tomato plants. Ps—pollen sac; cr—crystal of calcium oxalate; cw—cell wall; crp—crystal-bearing parenchymatous cell; t—tapetum; ml—middle layer cell. Bar 2 μm.

The space between the vascular bundle, sacs and epidermis is filled with several layers of parenchymatous cells. These cells also separate two pollen sacs within the same theca. At the stage of tetrads formation in the parenchymatous tissue adjacent to the outer part of the anther, a separate group of cells is stand out called crystal-bearing cells (Figure S1a,b), due to the fact that intensive deposition of calcium oxalate crystals begins in these cells. Crystals of various sizes were formed in these cells, usually having a stellate, with highly branched shapes (Figure 2). Normally, they usually form in two subepidermal layers of parenchymatous cells located between two pollen sacs of one theca (Figure S1a). As well as the idioblasts of the leaf parenchyma, the crystal-bearing cells of parenchymatous connective tissue of anther remained viable and were destroyed during the formation of large crystals in the vacuole (Figure 3 and Figure 4).

**Figure 5:**
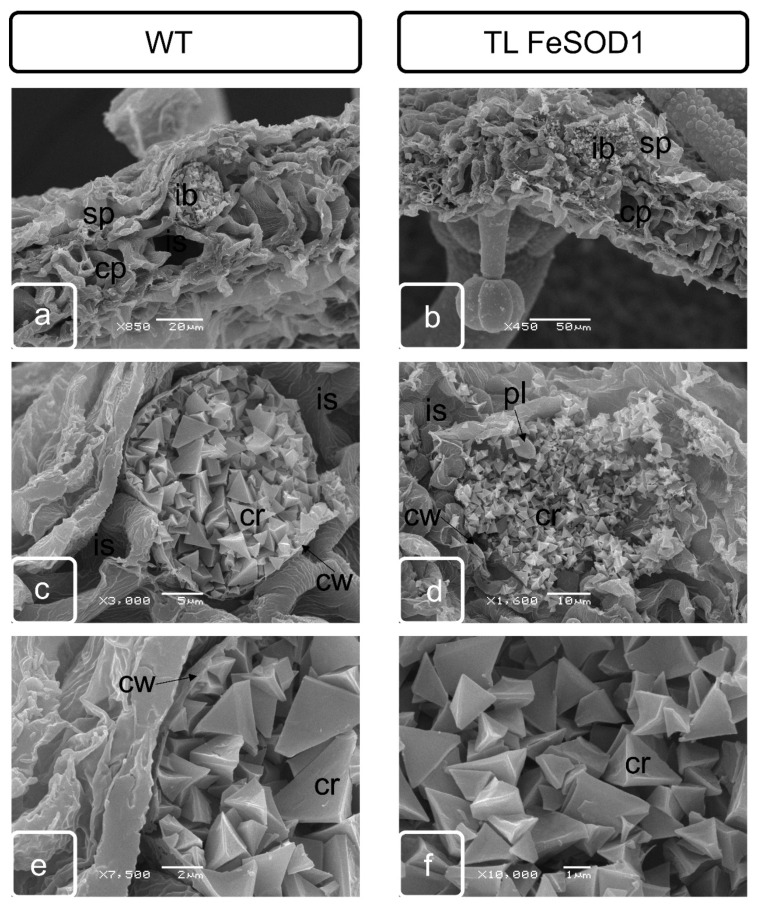
Scanning electron microscopy of spongy parenchyma cells of leaves of WT (**a**,**c**,**e**,**g**) and transgenic plants (**b**,**d**,**f**,**h**) from young (**a**–**d**) and mature (**e**–**h**) leaves. Ib—idioblast; sp—spongy parenchyma cells; cp—columnar parenchyma cells; cr—crystals of calcium oxalate; cw—cell wall; pl—plastids; is—intercellular space.

At the final stage, organelle damage, cytoplasm compaction and subsequent lysis of the cell compartment were observed (Figure 3e,g and Figure 4e,g). This was accompanied by a modification of the cell wall; it became thinner and had a deformed structure (Figure 5). As the anther developed, the cell walls of the crystal-bearing parenchymatous cells were destroyed, forming a space filled with numerous druses of calcium oxalate (Figure 3e,g and Figure 4e,g). Then the barrier of connective tissue between pollen sacs is disintegrated, uniting adjacent sacs into one cavity. Simultaneously with these processes, changes were occurred in the adjacent outer tissues of the anther. At the initial stage, small cells of the monolayer epidermis, with the exception of paired cells directly corresponding to the stomium, where the rupture later occurs, began to grow. They bulged outward while maintaining surface area, and formed ridge-like thickened formations (Figure S1c–f). These cells have been covered with cuticles, which ensured the strength of this structure. In normally developing anthers of tomato, under the epidermis, there is a fibrous layer of the endothecium consisting mainly of one layer of cells.

Thus, when opening the anther of fertile tomato form, the outer part is represented by two layers of modified cells of the epidermis and endothecium (Figure S1c,e), and the rupture is probably provided by tension in the ridge zones and interaction of two remaining unmodified epidermal cells with calcium oxalate druses, located in the zone of sacs connection. Modifications to the tomato flower due to ectopic expression of a foreign enzyme can lead to significant disturbances. FeSOD functioning caused a decrease in both the total height of the flower and all its components, such as petals, perianth, and anthers. However, this did not prevent the development of the female gametophyte, since the plants were capable of producing fruits. Most of the fruits either did not contain seeds at all, or the number of seeds was single that indicated impaired fertility before the start of the study. Cell formation and modification processes and interactions appear to be of paramount importance in the formation of fertile pollen (Figure 1, Figure S1). Thus, pollen grains forming at disturbance of anther development in transgenic tomato line, which expressed the Fe-dependent superoxide dismutase, had noticeable violations both in the surface structure and in shape and, mostly, had more or less significant deformations (Figure 1f,h and Figure 3b,d)

### 2.2. Features of the Ultrastructure of Pollen Sacs and Crystal-Bearing Parenchymatous Cells of the Connective Tissue

By the end of the formation of full-fledged pollen, the bordering tissues of the pollen sacs undergo significant modification. Tapetum is practically destroyed, as a result of the destruction of the crystal-bearing parenchyma, crystals of calcium oxalate can be found in it. Cell walls undergo modifications and either partially degrade or become thinner. At this stage, crystal-bearing cells were partially destroyed and can be preserved only in the lower part of the anther (Figure 2). Crystal formation is delayed and cell destruction does not occur in transgenic plants with impaired fertility (Figure 3).

### 2.3. Morphological Features of Anthers of FeSOD-Transgenic and WT Tomato Plants

At the stage preceding the opening of the anther in fertile wild-type plants, it is possible to observe a small zone of the location of the crystals oxalate druses in the partially destroyed cells of the crystal-bearing parenchyma of the upper part of the anther, where its opening is initiated (Figure 2e,g). In transgenic plants, the cells do not have a pronounced localization. Most of the cells retain their internal structure and cell walls, and the number of such cells is much greater than in fertile plants. The druses remain attached to cell membranes, which indicates blocking of cell death and lysis (Figure 2d,f,h).

In the cells adjacent to the rupture site in the lower part of the anthers in WT plants, drusen line the rupture surface (Figure 3e,g), while in the anther cells of the transgenic line, the deposition of oxalates and ruptures of the cells of the adjacent tissues of the endothetium and parenchyma are blocked (Figure 3d,f,h).

Significant differences can be observed in the lower part of the anther. Although the shape and amount of calcium oxalate crystals in the WT plants were slightly changed in this zone, the zone is clearly localized and connects two sacs of the one theca (Figure 3a,c,e,g). In transgenic plants with impaired fertility, living parenchymatous cells with extant cytoplasm were located between two pollen sacs; druse formation could not be identified (Figure 3b,d,f,h). 

Changes in cell wall morphology (formation of cavities) were observed in tapetum and crystal-bearing tissues of WT tomato plants (Figure 4). No cell wall modifications were noted in anthers of transgenic plants at the same stage.

The distribution of oxalate crystals and their shape were different in the upper and middle parts of the anther of WT plants, which corresponds to the data of scanning microscopy (Figure 2 and Figure 3). In the tapetum and parenchyma tissues of the lower part of the anthers of transgenic plants, the formation of crystals was significantly reduced (Figure 3 and Figure 4), while in the upper part of the anthers, the synthesis of crystals remained unchanged (Figure 2 and Figure 4).

### 2.4. Comparative Study of Idioblasts of WT and FeSOD-Transgenic Plants

The formation of specialized cells (idioblasts), which accumulate calcium oxalate crystals, in the parenchyma of photosynthetic tissues is characteristic of tomato, like other plants of the *Solanaceae* family. These cells are located in the spongy mesophyll and differ significantly from neighboring cells both in the ultrastructure of cytoplasm and organelles. Thus, if plastids in neighboring cells have a developed structure of lamellae, granae, inherent of photosynthetic tissues, and often contain significant deposits of starch, then plastids in idioblasts have a more rounded shape and a small number of membrane structures, single granae and lamellae (Figure 5). At the initial stage, no significant differences in idioblast structure were observed between the WT and FeSOD-transgenic plants. In mature leaves of WT plants, the shape of the crystal-bearing cells remained regular, rounded, the cell wall retained its structure, although it was somewhat thinner (Figure 5e,g), while in FeSOD-transgenic plants the cells often had an irregular shape and a damaged membrane (Figure 5f,h).

### 2.5. Transmission Microscopy of Cross Sections of Idioblasts Containing Spongy Parenchyma Tissue from Mature Leaf of Tomato Plants

**Figure 6:**
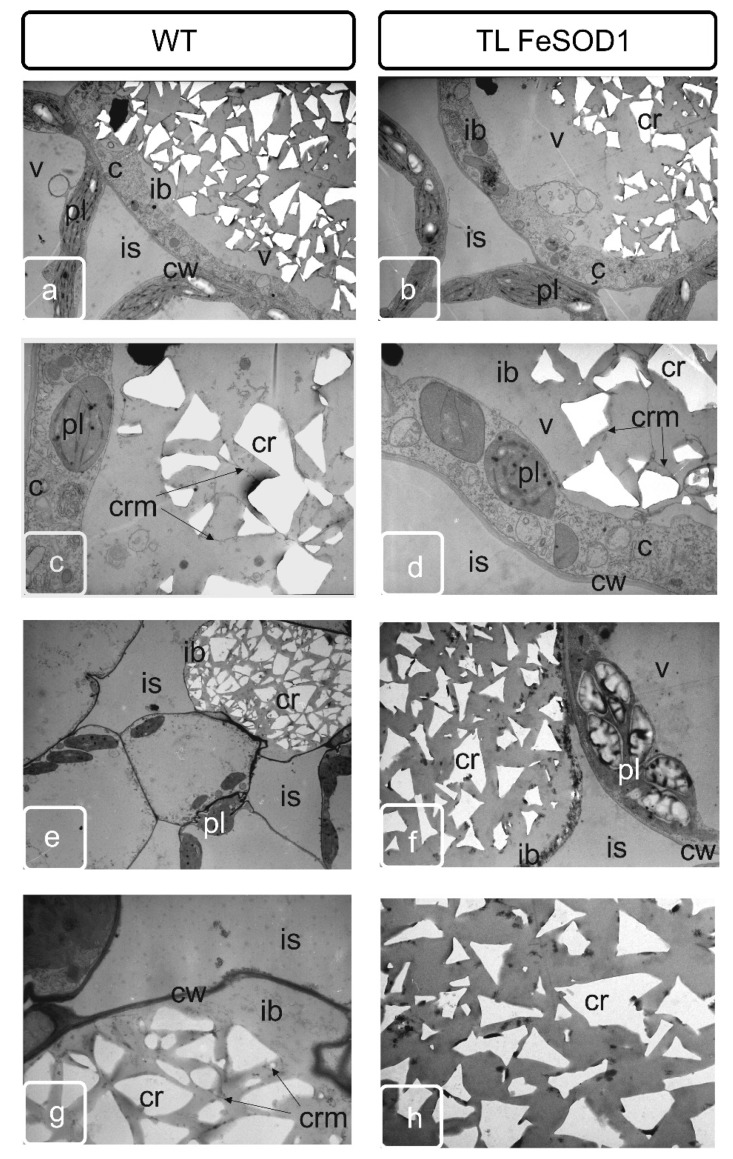
Transmission electron microscopy of leaf parenchyma idioblasts from WT (**a**,**c**,**e**,**g**) and transgenic tomato plants (**b**,**d**,**f**,**h**) in the early (**a**–**d**) and later stages of formation (**e**–**h**). Ib—idioblast; cr—oxalate calcium crystal; crm—crystal chamber membrane; c—cytoplasm; is—intercellular space; cw—cell wall; v—vacuole; pl—plastid.

In both WT and transgenic plants, idioblasts were located in the zone adjacent to the lower epidermis of spongy parenchyma (Figure 6). The cells contained a significant amount of prismatic calcium oxalate inclusions of various sizes in mature leaves. Idioblasts in the WT leaf had a more regular shape and contained large crystalline grains in the central part, and somewhat smaller ones closer to the periphery (Figure 6a,c,e). The idioblasts in the leaf of FeSOD-transgenic plant were larger, had an irregular shape and contained inclusions of smaller crystals of the same size (Figure 6b,d,f), corresponding in shape to the prismatic structure of WT plant ideoblasts (Figure 6e).

Various forms of calcium oxalate crystals such as prismatic, needle-like, druses, raphides are present in different tomato tissues, and are sometimes found in cells, uncharacteristic for crystal formation (Figure S2).

## 3. Discussion

Programed cell death is one of the most important processes that ensure the formation and successive development of specialized organs and tissues at different stages of ontogenesis [35]. Disruption of the mechanisms of the specialized cells formation associated with a delay or acceleration of the completion of processes—which provide local detoxification as a result of macro- and micro-autophagy or various types of program cell death—can significantly change the phenotype of plants, causing obvious morphological changes in some organs and tissues [36]. Changes in morphology, in turn, cause disruption of metabolites and water movement and can interfere with the normal process of metabolism in plant, ensuring its resistance to unfavorable environmental conditions, growth, development and/or reproduction. Anther dehiscence is one of the most important processes providing generative reproduction of higher plants. This process is provided by two successive transformations in the anther cells. First of all, individual cells in the connective parenchyma tissue located between two pollen sacs in the theca are modified into specialized crystal-bearing cells. Further, the cells of the epidermis, which are adjacent to the crystal-bearing cells in the barrier between the sacs, form a ridge obviously protrusive on the surface of the epidermis. The ridge consists of rows of flattened cells along the future rupture, probably providing the necessary mechanical tension. The forming crystals of calcium oxalate in anther cells contain large druses, in contrast to crystal-forming mesophyll cells, leaf idioblasts, which are characterized by prismatic crystals. Although both have a clear adhesion to membranes that are clearly identified when the sections of such cells are analyzed by transmission microscopy, this relationship was not obvious by scanning microscopy. In our study, it can be seen that until the destruction of cells, the druses are unambiguously attached to the cell matrix, remaining connected to the surface of the cell walls even at a sufficient distance (Figure 2, Figure 3 and Figure 4).

However, it has not been possible to reveal clear patterns of crystal attachment in idioblasts (Figure 5 and Figure 6). The fact that the crystals remain inside the cell wall of the idioblast indicates the presence of a material that ensures and maintains the stability of the given intracellular structure, preventing release. This indicates the preservation of the membrane chambers, which ensure the primary formation of crystals and the optimal conditions for crystallization of calcium oxalate necessary for this process [37,38]. These membrane structures were retained even with the loss of the cytoplasmic compartments of the idioblast (Figure 5 and Figure 6). In a number of studies, the assumption was experimentally confirmed that crystal-bearing cells perform the function of removing excess calcium from the apoplast of surrounding cells [39,40]. Although both have an obvious adhesion to membranes that are clearly identified when the sections of such cells are analyzed by transmission microscopy; and this relationship was not obvious by scanning microscopy. Precursor of oxalic acid in idioblasts was found to be ascorbic acid, but not glycolate [2,41,42]. In contrast to assumptions of a number of early studies, the insufficient development of plastids in the idioblasts of both WT and transgenic plants indicates in favor of the supply of initial material from the surrounding cells of the photosynthetic parenchyma, and not as a result of glycolate biosynthesis in cell (Figure 5) [43,44]

Differences between transgenic and WT plants were identified in both generative and vegetative tissues (Figure 2, Figure 3, Figure 4 and Figure 5).

Disturbances in oxalate biosynthesis processes as a result of increased superoxide dismutase activity in transgenic plants were accompanied by perturbations in the development of flower organs and, in particular, the machinery that ensures the junction of pollen sacs in theca, and subsequent dehiscence of the anthers. It can be assumed that the deformation of the anthers and a decrease in their size leads to a disturbance in the flow of assimilates. Probably, this also causes disturbances in the distribution of the excess pool of calcium and oxalic acid, which are capable to initiate specialized membrane structures in the vacuolar compartment [37]. As a result, in the upper part of the transgenic plant anther, not two layers of specialized crystal-bearing cells adjacent to the stomium were formed as in the WT plants, but more. The number of cell layers in this zone was clearly increased (Figure 2 and Figure 3; Figure S1). In addition, the formation of separate crystals in the cells of the adjacent connective parenchyma tissue could be noted (Figure 2 and Figure 4).

It should also be noted that in the lower part of the anthers, where the formation of crystals is reduced and they are smaller in size, the similar process is impaired in transgenic plants and cannot provide not only normal junction of pollen sacs, but also does not provide full-fledged dehiscence (Figure 3 and Figure 4). In our opinion, it is the development of the crystal-bearing cells that becomes the crucial issue and prevents the normal pollination in FeSOD-transgenic plants. A similar point of view was claimed by a number of authors as a result of studying other tomato plants with impaired fertility [16].

We have previously shown that tomato plants with this gene had nonspecific resistance to late blight [43]. However, the role of oxalates in injury of fertility has not been investigated in this study.

The differences revealed in this work between idioblasts in WT and transgenic plant (Figure 5 and Figure 6) may also have been implicated in resistance to pathogens. Such examples, demonstrating the role of calcium oxalate crystals, are widely discussed in the works on the pathogenesis of plants with idioblasts [4,45]. The change in shape and increase in size of these crystal-bearing cells is accompanied by a decrease in the size of prismatic crystals. This may be a reflection of changes in development of the membrane system, which ensure the scavenging of excess calcium using oxalic acid, and the oxalate supply into the membrane chamber for crystallization [2]. Changing the crystal-bearing cell can be significant for other functions as well. Thus, the probable use of oxalates as a mobile calcium reserve by plant cells is recently discussed [46], previously considered as a final product that is not subject to further metabolization [2,47].

The present work allows us to confirm the role of calcium oxalate and oxalate containing cells in ensuring the fertility of tomato [16] and other crops [48]. It can also be assumed that an excess activity of superoxide dismutase causes a clear change in metabolite fluxes, which we detected when the formation of starch grains in columella cells is disrupted [46]. In this study, we have shown that programed cell death of the crystal-bearing parenchyma is a sensitive mechanism that ensures reliable anther dehiscence and subsequent self-pollination. This PCD depends on metabolic transformations associated with the scavenging of ROS, leading to a change in the localization and deposition of oxalates in parenchymal cells adjacent to the stomium. It would be important to identify this type of cell death [35], and to reveal what is the reason for the disturbance of the exact localization of oxalates in plants with reduced fertility.

## 4. Materials and Methods

### 4.1. Plant Material

The in vitro cultivated tomato (*Solanum lycopersicum* L.) plants of the control cultivar Belyi Naliv (wild type—WT) and transgenic line 6 expressing the superoxide dismutase FeSOD gene from *Arabidopsis thaliana* (L.) Heynh. were the object of comparative study. The line 6, like some other lines (characterized by high levels of superoxide dismutase activity, but not other antioxidant enzymes) was characterized by increased tolerance to pathogens, changes in the ultrastructural organization of plastids and mitochondria [30], as well as problems with seed formation due to the shedding of flowers and the formation of low-seeded or parthenocarpic fruits. Cloned tomato plants were grown in a growth chamber for 14 days at 25 ± 2 °C under 16/8 h light/dark diurnal cycle with a light intensity of 160 µmol photons m^−2^ s^−1^, and air humidity of 60–70% in plastic container filled with sterilized soil. After that, pieces of plant leaf, anthers and flowers were fixed for various microscopic analyses.

### 4.2. Sampling for Scanning and Transmission Electron Microscopy

Leaves, flowers, and anthers of mature plants in the generative stage (100 days after sowing) were used for the analysis. The plants had 2–3 inflorescences. The lower inflorescences had 1–3 fruits that began to form. For fixation, a 2–3 mm^2^ fragment of the central part of the upper leaves located in the middle part of plant was used. Flowers and anthers were placed in the fixator entirely. For fixation, 2% glutaraldehyde based on 0.1 M Na-phosphate buffer (pH 7.4) was used with the addition of 1.5% sucrose. The samples were washed twice with the same buffer and: (1) they were dehydrated in a series of alcohols for scanning microscopy, dried and sprayed (Section 4.3), or (2) they were postfixed with a 1% OsO_4_ solution, followed by washing, dehydration, and encapsulation in resin according to the method described in Section 4.4.

### 4.3. Sample Preparation for Scanning Microscopy

The samples were fixed in a 2.5% glutaraldehyde in 0.1 M Sorenson buffer, pH 7.2 and were washed in buffer. The samples were dehydrated through ethanol series (30% 30′, 50% 30′, 70% 30′, 96% 30′, 2 × 100% 30′). Then CO_2_ was added for the sample to be critical-point-dryer with a Hitachi HCP-2 critical point dryer (Hitachi, Tokyo, Japan) was applied. Then, the dry seeds were mounted on a SEM stub with carbon conductive tabs and coated with gold and palladium using an Eiko IB-3 ion-coater (Eiko, Tokyo, Japan). The samples were then observed under a JSM-6380LA SEM (JEOL, Tokyo, Japan) and a Camscan-S2 SEM (Cambridge Instruments, Cambridge, UK) in the Laboratory of Electron Microscopy (Biological Faculty of Lomonosov Moscow State University).

Flowers, anther and leaf fragments were studied by scanning electron microscope JEOL, JSM-6380LA, accelerating voltage 20 kV; IB-3 Ion Coater (EIKO, Shawnee, KS, USA), the thickness of Au layer was 20 nm.

To obtain anther surface and slice photos, transverse sections of leaf from five plants were placed onto an SEM stub, which is covered by carbon adhesive tape (Double-Sided Carbon Tape, 8 × 20 mm, EMS Cat#77817-08-AL, Shanghai, China). Magnifications ×50–×2000 were used. The samples were determined using the SEM Control User Interface Version 7.11 Copyright 2004 JEOL Technics LTD. Anthers from three flowers from I order inflorescences was taken in five plants and transgenic line 6. These samples was placed on a stub onto disks from SPI Supplies Division of Structure Probe, Inc., West Chester, PA, USA.

### 4.4. Sample Preparation for Transmission Electron Microscopy

Leaf, anthers and flowers samples were fixed for 24 h at +4 °C in 2% glutaraldehyde solution on a 0.1 M Na-phosphate buffer (pH 7.2) with sucrose (15 mg/mL), and then two times rinsed with buffer. Postfixation of the material was carried out in a 1% aqueous solution of OsO_4_. Dehydration with ethyl alcohol and the embedding of the material in epoxy resin were carried out according to the standard procedure. The polymerization was carried out in two stages (day +34 °C, day +60 °C).

The anthers ultra-thin sections were stained with uranyl acetate and lead citrate and examined using a H-500 electron microscope (Hitachi, Tokyo, Japan).

## 5. Conclusions

The deposition of calcium oxalates in specialized crystal-bearing cells plays a significant role in anther dehiscence and in ensuring fertility, which, when analyzing the state of crystal-bearing tissue in the upper and lower parts of the anther, can be an additional parameter for preliminary assessment of the fertility of mutants, hybrid lines, and transgenic plants with parthenocarpic or low-seeded fruits. Although we have been able to establish a probable relationship between superoxide dismutase functioning and abnormalities in calcium oxalate formation, the mechanisms underlying this relationship remain to be determined.

## Supplementary Materials

The following are available online at https://www.mdpi.com/1422-0067/21/24/9480/s1, Figure S1: Structure of anther tissues in cross sections from tomato plants. Fragments of anthers with pollen sac (ps) of WT (a) and transgenic (b) plants, and cells of the forming crystal-bearing tissue. Fragments of an opening anther at the stage of the pollen formation completion, and changes in the morphology of ridges at the border of the stomium (c—WT, d—transgenic plants). Anther fragments at the stage preceding dehiscence (e—WT, f—transgenic plants), Figure S2: Structure (transmission transmission microscopy) of calcium oxalate crystals in tomato cells (top row shows a cell fragment, the row below shows an enlarged fragment containing crystals). Prismatic crystal in leaf idioblasts; druse in the vacuole of anther crystal-bearing cells; a complex of aggregate crystals (crystalline sand) in parenchymatous cells located next to the tapetum at the last stage of pollen formation; needle-like crystals in the cells of the leaf epidermis, raphide crystal chambers in the central vacuole of the anther parenchyma, raphids in the vacuoles of photosynthetic cells of the leaf parenchyma.

## References

[1] White P.J., Broadley M.R. (2003). Calcium in plants. Ann. Bot..

[2] Franceschi V.R., Nakata P.A. (2005). Calcium oxalate in plants: Formation and function. Annu. Rev. Plant Biol..

[3] Prasad R., Shivay Y.S. (2017). Oxalic acid/oxalates in plants: From self-defence to phytoremediation. Curr. Sci..

[4] Libert B., Franceschi V.P. (1987). Oxalate in crop plants. J. Agric. Food Chem..

[5] Franceschi V.R., Horner H.T. (1980). Calcium oxalate crystals in plants. Bot. Rev..

[6] McLaughlin S.B., Wimmer R. (1999). Tansley review no. 104: Calcium physiology and terrestrial ecosystem processes. New Phytol..

[7] Hara T., Kobayashi E., Ohtsubo K., Kumada S., Kanazawa M., Abe T., Itoh R.D., Fujiwara M.T. (2015). Organ-level analysis of idioblast patterning in *Egeria densa* Planch. leaves. PLoS ONE.

[8] Cuellar-Cruz M., Perez K.S., Mendoza M.E., Moreno A. (2020). Biocrystals in plants: A short review on biomineralization processes and the role of phototropins into the uptake of calcium. Crystals.

[9] Marina M., Romero F.M., Villarreal N.M., Medina A.J., Gárriz A., Rossi F.R., Martinez G.A., Pieckenstain F.L. (2019). Mechanisms of plant protection against two oxalate-producing fungal pathogens by oxalotrophic strains of *Stenotrophomonas* spp.. Plant Mol. Biol..

[10] Mazen A.M.A., El Maghraby O.M.O. (1998). Accumulation of cadmium, lead and strontium, and a role of calcium oxalate in water hyacinth tolerance. Biol. Plant..

[11] Mazen A.M.A. (2004). Calcium oxalate deposits in leaves of *Corchorus olitotius* as related to accumulation of toxic metals. Russ. J. Plant Physiol..

[12] Leitenmaier B., Küpper H. (2013). Compartmentation and complexation of metals in hyperaccumulator plants. Front. Plant Sci..

[13] Korth K.L., Doege S.J., Park S.-H., Goggin F.L., Wang Q., Gomez S.K., Liu G., Jia L., Nakata P.A. (2006). *Medicago truncatula* mutants demonstrate the role of plant calcium oxalate crystals as an effective defense against chewing insects. Plant Physiol..

[14] Rahman M.M., Abdullah R.B., Wan Khadijah W.E. (2013). A review of oxalate poisoning in domestic animals: Tolerance and performance aspects. J. Anim. Physiol. Anim. Nutr..

[15] Helper P.K., Wayne R.O. (1985). Calcium and plant development. Annu. Rev. Plant Physiol..

[16] Oryol L.I., Zhakova M.A. (1977). The mechanism of anther dehiscence of tomato *Lycopersicon esculentum* Mill. (*Solanaceae*). Bot. Zhurnal.

[17] Bonner L.J., Dickinson H.G. (1989). Anther dehiscence in *Lycopersicon esculentum* Mill. I. Structural aspects. New Phytol..

[18] Pacini E. (2010). Relationships between tapetum, loculus, and pollen during development. Int. J. Plant Sci..

[19] Burrieza H.P., López-Fernández M.P., Láinez V., Montenegro T., Maldonado S. (2010). On the nature and origin of the oxalate package in *Solanum sisymbriifolium* anthers. Protoplasma.

[20] Wilson Z.A., Song J., Taylor B., Yang C. (2011). The final split: The regulation of anther dehiscence. J. Exp. Bot..

[21] Burchi G., Bauchan G.R., Murphy C., Roh M.S. (2014). Characterization of calcium crystals in *Abelia* spp. using X-ray diffraction and electron microscopy. J. Hort. Sci. Biotech..

[22] Du M., Zhou K., Liu Y., Deng L., Zhang X., Lin L., Zhou M., Zhao W., Wen C.-L., Xing J. (2020). A biotechnology-based male-sterility system for hybrid seed production in tomato. Plant J..

[23] van Ginkel M., Flipphi R.C. (2020). Why self-fertilizing plants still exist in wild populations: Diversity assurance through stress-induced male sterility may promote selective outcrossing and recombination. Agronomy.

[24] Carroll T.W., Mayhew D.E. (1976). Anther and pollen infection in relation to the pollen and seed transmissibility of two strains of barley stripe mosaic virus in barley. Can. J. Bot..

[25] Hunter D.G., Bowyer J.W. (1997). Cytopathology of developing anthers and pollen mother cells from lettuce plants infected by lettuce mosaic potyvirus. J. Phytopathol..

[26] Stikić R., Jovanović Z., Vucelić-Radović B., Marjanović M., Savić S. (2015). Tomato: A model species for fruit growth and development studies. Bot. Serbica.

[27] Bohner J., Bangerth F. (1988). Cell number, cell size and hormone level in semi-isogenic mutants of *Lycopersicon pimpinellifolium* differing in fruit size. Physiol. Plant..

[28] Pesaresi P., Mizzotti C., Colombo M., Masiero S. (2014). Genetic regulation and structural changes during tomato fruit development and ripening. Front. Plant Sci..

[29] Gorguet B., Van Heusden A.W., Lindhout P. (2005). Parthenocarpic fruit development in tomato. Plant Biol..

[30] Serenko E.K., Baranova E.N., Balakhnina T.I., Kurenina L.V., Gulevich A.A., Kosobruhov A.A., Maysurian A.N., Polyakov V.Y. (2011). Structural organization of chloroplast of tomato plants *Solanum lycopersicum* transformed by Fe-containing superoxide dismutase. Biochem. (Mosc.) Suppl. Ser. A Membr. Cell Biol..

[31] Ekici N., Dane F. (2007). Calcium oxalate crystals in vegetative and floral organs of *Galanthus* sp. (*Amaryllidaceae*). Asian J. Plant Sci..

[32] Ekici N., Feruza D.A.N.E. (2009). Calcium oxalate crystals during development of male and female gametophyte in *Leucojum aestivum* (*Amaryllidaceae*). J. Appl. Biol. Sci..

[33] Baranova E.N., Kurenina L.V., Smirnov A.N., Beloshapkina O.O., Gulevich A.A. (2017). Formation of the hypersensitivity response due to the expression of FeSOD1 gene in tomato when it is inoculated with *Phytophthora infestans*. Russ. Agric. Sci..

[34] Bogoutdinova L.R., Lazareva E.M., Chaban I.A., Kononenko N.V., Dilovarova T.A., Khaliluev M.R., Kurenina L.V., Gulevich A.A., Smirnova E.A., Baranova E.N. (2020). Salt stress-induced structural changes are mitigated in transgenic tomato plants over-expressing superoxide dismutase. Biology.

[35] Van Doorn W.G., Beers E.P., Dangl J.L., Franklin-Tong V.E., Gallois P., Hara-Nishimura I., Jones A.M., Kawai-Yamada M., Lam E., Mundy J. (2011). Morphological classification of plant cell deaths. Cell Death Diff..

[36] Khaliluev M.R., Chaban I.A., Kononenko N.V., Baranova E.N., Dolgov S.V., Kharchenko P.N., Polyakov V.Y. (2014). Abnormal floral meristem development in transgenic tomato plants do not depend on the expression of genes encoding defense-related PR-proteins and antimicrobial peptides. Rus. J. Dev. Biol..

[37] Katayama H., Fujibayashi S., Sugimura Y. (2007). Cell wall sheath surrounding calcium oxalate crystals in mulberry idioblasts. Protoplasma.

[38] Gębura J., Winiarczyk K. (2016). A study on calcium oxalate crystals in *Tinantia anomala* (*Commelinaceae*) with special reference to ultrastructural changes during anther development. J. Plant Res..

[39] Borchert R. (1986). Calcium acetate induces calcium uptake and formation of calcium-oxalate crystals in isolated leaflets of *Gleditsia triacanthos* L.. Planta.

[40] Fink S. (1991). Comparative microscopical studies on the patterns of calcium oxalate distribution in the needles of various conifer species. Plant Biol..

[41] Horner H.T., Kausch A.P., Wagner B.L. (2000). Ascorbic acid: A precursor of oxalate in crystal idioblasts of *Yucca torreyi* in liquid root culture. Int. J. Plant Sci..

[42] Kostman T.A., Tarlin N.M., Franceschi V.R. (2007). Autoradiography utilizing labeled ascorbic acid reveals biochemical and morphological details in diverse calcium oxalate crystal-forming species. Funct. Plant Biol..

[43] Arnott H.J., Pautard F.G.E. (1970). Calcification in Plants. Biological Calcification: Cellular and Molecular Aspects.

[44] Kausch A.P., Horner H.T. (1983). The development of mucilaginous raphide crystal idioblasts in young leaves of *Typha angustifolia* L. (*Typhaceae*). Am. J. Bot..

[45] Yang X., Yang J., Wang Y., He H., Niu L., Guo D., Xing G., Zhao Q., Zhong X., Sui L. (2019). Enhanced resistance to sclerotinia stem rot in transgenic soybean that overexpresses a wheat oxalate oxidase. Transgenic Res..

[46] Paiva E.A.S. (2019). Are calcium oxalate crystals a dynamic calcium store in plants?. New Phytol..

[47] Ledbetter M.C., Porter K.R. (2012). Introduction to the Fine Structure of Plant Cells.

[48] Meric C., Dane F. (2004). Calcium oxalate crystals in floral organs of *Helianthus annuus* L. and *H. tuberosus* L. (*Asteraceae*). Acta Biol. Szeged..

