# Supplementary material for: Possible Role of Crystal-Bearing Cells in Tomato Fertility and Formation of Seedless Fruits
Source: Int J Mol Sci. 2020 Dec 13;21(24):9480. doi: 10.3390/ijms21249480 (PMC7763322; doi:10.3390/ijms21249480)
Supplement: Supplementary file 1 [file ijms-21-09480-s001.pdf]

Article

Possible Role of Crystal-Bearing Cells in Tomato Fertility and Formation of Seedless Fruits

Ekaterina N. Baranova [1,2,*], Inna A. Chaban [2], Ludmila V. Kurenina [3], Ludmila N. Konovalova [1,2], Natalia V. Varlamova [3], Marat R. Khaliluev [3,4] and Alexander A. Gulevich [3,*]

[1] Plant Protection Laboratory, N.V. Tsitsin Main Botanical Garden of Russian Academy of Sciences, 127276 Moscow, Russia; konovalova-lu@yandex.ru

[2] Cell Biology Laboratory, All-Russian Scientific Research Institute of Agricultural Biotechnology, 127550 Moscow, Russia; inna_chaban@rambler.ru

[3] Plant Cell Engineering Laboratory, All-Russian Scientific Research Institute of Agricultural Biotechnology, 127550 Moscow, Russia; ludmila.kur2208@gmail.com (L.V.K.); nv_varlamova@rambler.ru (N.V.V.); marat131084@rambler.ru (M.R.K.)

[4] Agronomy and Biotechnology Faculty, Moscow Timiryazev Agricultural Academy, Russian State Agrarian University, Timiryazevskaya 49, 127550 Moscow, Russia

* Correspondence: greenpro2007@rambler.ru (E.N.B.); a_gulevich@mail.ru (A.A.G.); Tel.: +7-903-624-5971 (E.N.B.); +7-968-084-6878 (A.A.G.)

Table of content

**Figure S1.**	Structure of anther tissues in cross sections from tomato plants. Fragments of anthers with pollen sac (ps) of WT (a) and transgenic (b) plants, and cells of the forming crystal-bearing tissue. Fragments of an opening anther at the stage of the pollen formation completion, and changes in the morphology of ridges at the border of the stomium (c - WT, d - transgenic plants). Anther fragments at the stage preceding dehiscence (e - WT, f - transgenic plants).	S3
**Figure S2.**	Structure (transmission transmission microscopy) of calcium oxalate crystals in tomato cells (top row shows a cell fragment, the row below shows an enlarged fragment containing crystals). Prismatic crystal in leaf idioblasts; druse in the vacuole of anther crystal-bearing cells; a complex of aggregate crystals (crystalline sand) in parenchymatous cells located next to the tapetum at the last stage of pollen formation; needle-like crystals in the cells of the leaf epidermis, raphide crystal chambers in the central vacuole of the anther parenchyma, raphids in the vacuoles of photosynthetic cells of the leaf parenchyma.	S4

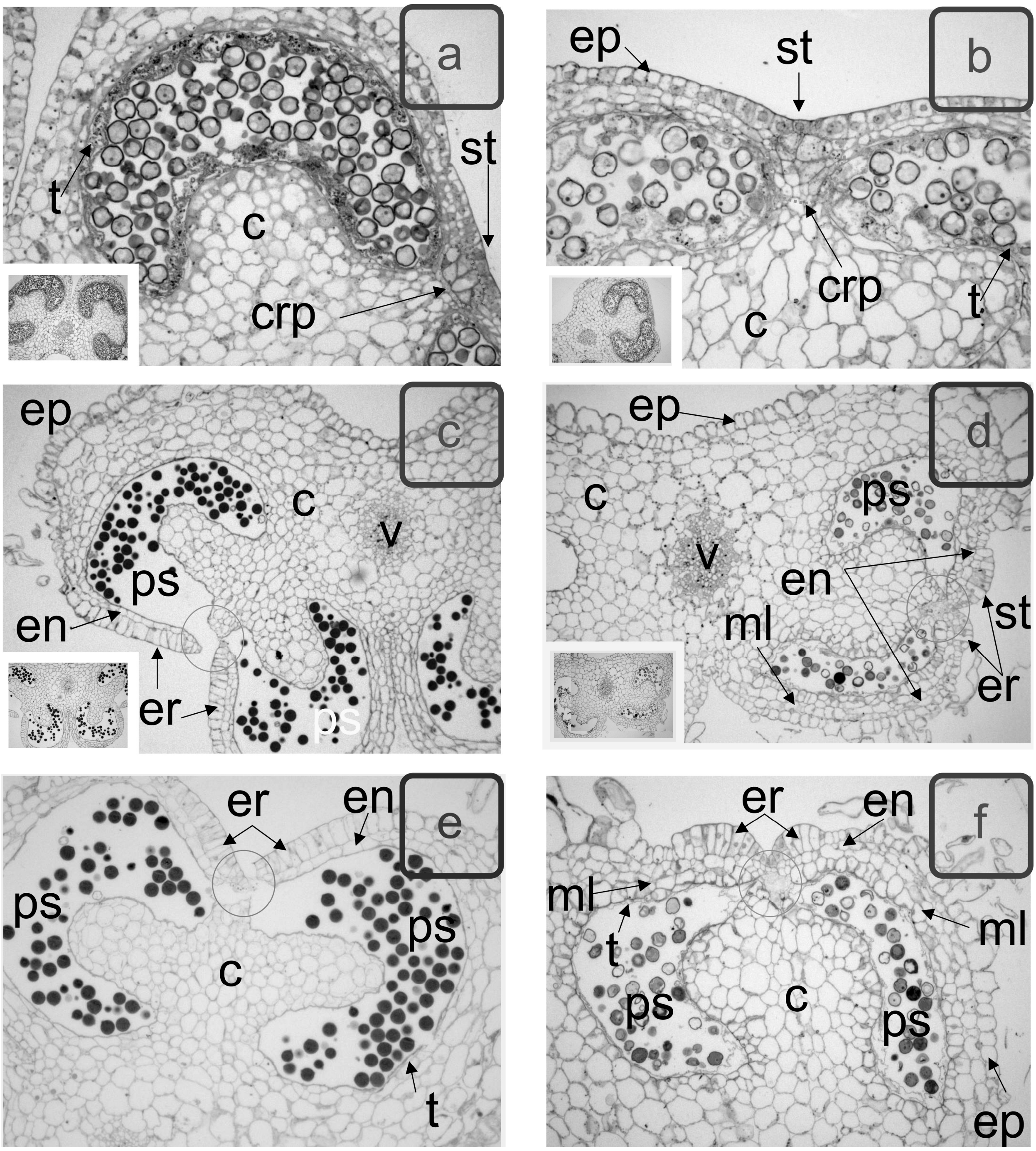

Figure S1. Structure of anther tissues in cross sections from tomato plants. Fragments of anthers with pollen sac (ps) of WT (a) and transgenic (b) plants, and cells of the forming crystal-bearing tissue. Fragments of an opening anther at the stage of the pollen formation completion, and changes in the morphology of ridges at the border of the stomium (c - WT, d - transgenic plants). Anther fragments at the stage preceding dehiscence (e - WT, f - transgenic plants). St - stomium; c - connective; v - vascular strand; crp - crystal-bearing parenchymatous cell, er - epidermal ridges, en - endothecium; ep - epidermal tissue; ml - middle layer cell; t - tapetum.

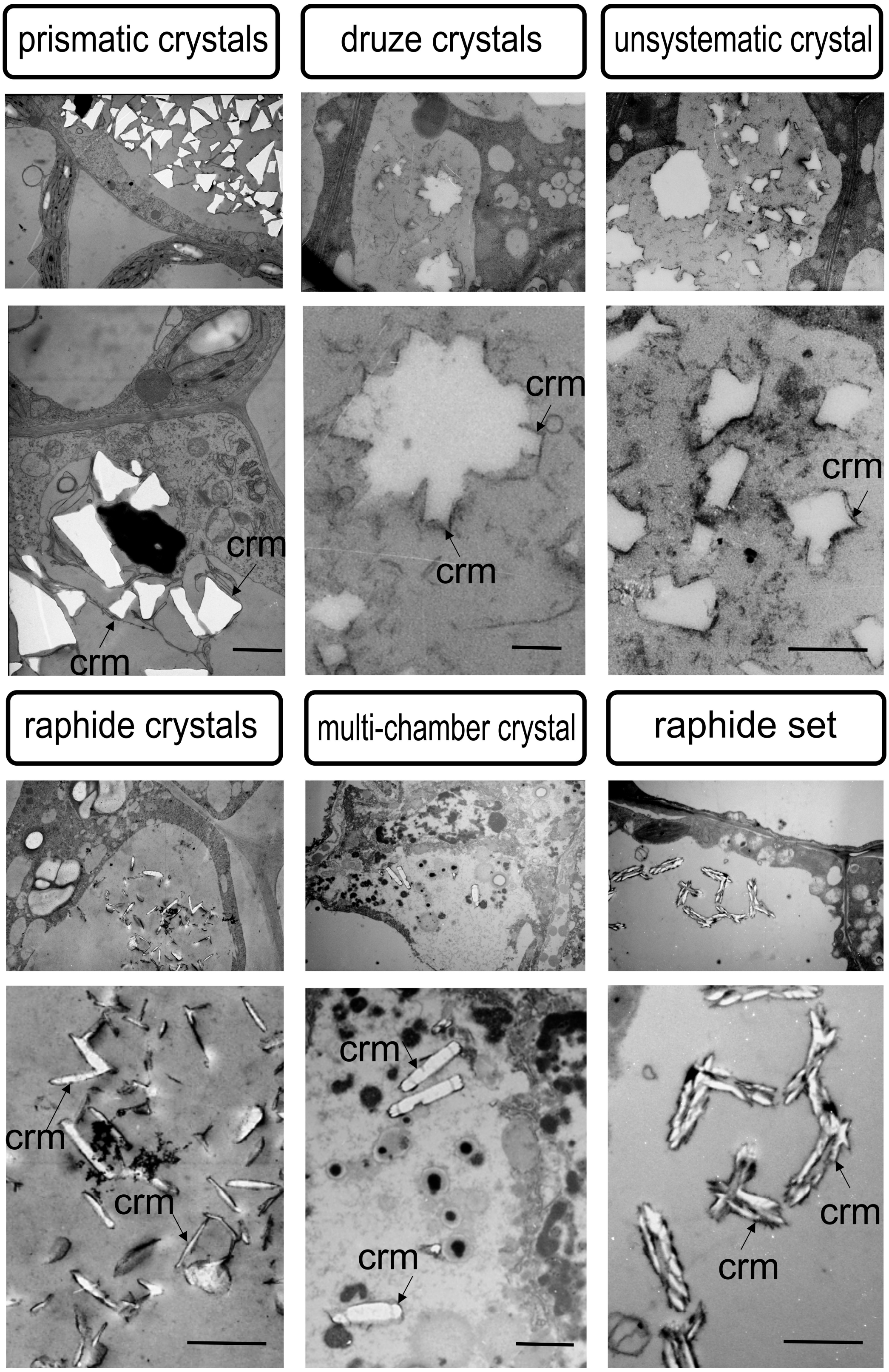

Figure S2. Structure (transmission microscopy) of calcium oxalate crystals in tomato cells (top row shows a cell fragment, the row below shows an enlarged fragment containing crystals). Prismatic crystal in leaf idioblasts; druse in the vacuole of anther crystal-bearing cells; a complex of aggregate crystals (crystalline sand) in parenchymatous cells located next to the tapetum at the last stage of pollen formation; needle-like crystals in the cells of the leaf epidermis, raphide crystal chambers in the central vacuole of the anther parenchyma, raphids in the vacuoles of photosynthetic cells of the leaf parenchyma. crm - crystal chamber membrane. Bar 2 μm.
